# *EnrichIntersect*: an R package for custom set enrichment analysis and interactive visualization of intersecting sets

**DOI:** 10.1093/bioadv/vbac073

**Published:** 2022-09-27

**Authors:** Zhi Zhao, Manuela Zucknick, Tero Aittokallio

**Affiliations:** Department of Cancer Genetics, Institute for Cancer Research, Oslo University Hospital, Oslo N-0310, Norway; Department of Biostatistics, Oslo Centre for Biostatistics and Epidemiology (OCBE), Faculty of Medicine, University of Oslo, Oslo N-0372, Norway; Department of Biostatistics, Oslo Centre for Biostatistics and Epidemiology (OCBE), Faculty of Medicine, University of Oslo, Oslo N-0372, Norway; Department of Cancer Genetics, Institute for Cancer Research, Oslo University Hospital, Oslo N-0310, Norway; Department of Biostatistics, Oslo Centre for Biostatistics and Epidemiology (OCBE), Faculty of Medicine, University of Oslo, Oslo N-0372, Norway; Institute for Molecular Medicine Finland (FIMM), HiLIFE, University of Helsinki, Helsinki FI-00014, Finland

## Abstract

**Summary:**

Enrichment analysis has been widely used to study whether predefined sets of genes or other molecular features are over-represented in a ranked list associated with a disease or other phenotype. However, computational tools that perform enrichment analysis and visualization are usually limited to predefined sets available from public databases. To make such analyses more flexible, we introduce an R package, *EnrichIntersect*, which enables enrichment analyses among any ranked features and user-defined custom sets. For interactive visualization of multiple covariates, such as genes or other features, which are associated with multiple phenotypes and multiple sample groups, such as drug responses in various cancer types, *EnrichIntersect* illustrates all associations at a glance, hence explicitly indicating intersecting covariates between multiple phenotypic variables and between multiple sample groups.

**Availability and implementation:**

The *EnrichIntersect* R package is available at https://CRAN.R-project.org/package=EnrichIntersect via an open-source MIT license. A package installation process is described on CRAN at https://cran.r-project.org/. A user-manual description of features and function calls can be found from the vignette of our package on CRAN.

## 1 Introduction

Gene set enrichment or over-representation analysis maps large lists of genes into a smaller list of more easily interpretable gene sets or pathways (Subramanian *et al.*, 2005). However, most enrichment tools use already-existing gene sets or biochemical pathway databases, for example, Gene Ontology (GO) and Kyoto Encyclopedia of Genes and Genomes (KEGG). This limits the users when exploring their gene lists or other feature lists with respect to predefined sets of interest. Innis *et al.* (2021) provide an R package for customizing gene set enrichment analysis; however, this method identifies differentially expressed genes from a given data matrix with respect to only a single binary phenotype and subsequently performs enrichment analysis for the identified genes involved in customized gene set libraries, but there is no standalone function for the enrichment analysis in the later step. For wider applications, there is a need to implement computational tools that enable standalone enrichment analysis for any feature list of interest, based on user-defined custom sets. For instance, Zhao *et al.* (2022) analyzed tissue-specific drugs as drug classes enriched among a ranked drug list corresponding to a given cancer tissue. To enable more flexible enrichment analyses among any ranked features and user-defined custom set, we introduce the R (R Core Team, 2021) package *EnrichIntersect* for custom set enrichment analysis (CSEA).

The R package *EnrichIntersect* can also perform interactive visualization of the feature list of interest associated with multiple phenotypes and multiple sample groups using *ggplot2* (Wickham, 2016). Previously, Caldas *et al.* (2009) visualized a topic model via a so-called eye diagram, which is applicable to several latent variables (e.g. topics), many analysis levels (e.g. experiments), and a large number of tasks (e.g. gene sets). However, when there are many intermediate latent variables, such as genomic variables as covariates, our *EnrichIntersect* package makes it easier to visualize their relationships at multiple levels and with respect to multiple tasks using R package *networkD3* (Allaire *et al.*, 2017). For example, Zhao *et al.* (2022) developed cancer tissue-specific variable selection for large-scale drug screens, where a Sankey diagram, which is implemented in the *EnrichIntersect* package, was used to visualize the relationships between multiple genomic features and several drugs of interest (i.e. multiple tasks), corresponding to several cancer tissue types (i.e. sample groups). A similar alluvial plot was utilized by Gálvez *et al.* (2019) to visualize common GO terms enriched among clusters of self-organizing maps and to highlight response-significant modules from weighted gene correlation network analysis, but without providing publicly available software implementation.

## 2 Methods

### 2.1 Enrichment analysis

The methodology of CSEA is based on the guideline provided by Reimand *et al.* (2019). For a feature list with rank metric $s_{L}\geq \cdots \geq s_{i}\geq \cdots \geq s_{1}$, consider a user-defined feature set consisting of a list of *L_k_* features $G_{k}=\{g_{kj}:j=1,\ldots ,L_{k}\}$ indexed by *k* where each feature *g_kj_* must be represented in the ranked list. Define the set of features outside of *G_k_* as ${\bar{G}}_{k}=\{{\bar{g}}_{kj}:j=1,\ldots ,L-L_{k}\}$. A (weighted) Kolmogorov–Smirnov statistic can measure the enrichment score for a given feature set *G_k_*, i.e.

$$S_{k}=\underset{1\leq i\leq L}{\text{sup}}|F_{i}^{G_{k}}-F_{i}^{{\bar{G}}_{k}}|,$$

where $F_{i}^{G_{k}}$ and $F_{i}^{{\bar{G}}_{k}}$ are weighted empirical cumulative distribution functions

$$\begin{array}{c} F_{i}^{G_{k}}=\frac{\sum_{t=1}^{i}|s_{t}{|}^{\alpha }\;\cdot \;1_{\left\{{\text{feature}}_{t}\in G_{k}\right\}}}{\sum_{t=1}^{L}|s_{t}{|}^{\alpha }\;\cdot \;1_{\left\{{\text{feature}}_{t}\in G_{k}\right\}}},\\ F_{i}^{{\bar{G}}_{k}}=\frac{\sum_{t=1}^{i}|s_{t}{|}^{\alpha }\;\cdot \;1_{\left\{{\text{feature}}_{t}\in {\bar{G}}_{k}\right\}}}{L-L_{k}}, \end{array}$$

where *α* is for weighted enrichment score and $1_{\left\{\cdot \right\}}$ is the indicator function for membership in the specified feature list.

A large enrichment score *S_k_* indicates that the list of features is likely to be enriched to the given feature set *G_k_*. To determine the statistical significance of each enrichment score, permutation test is used to derive an empirical *P*-value. Accounting for the feature set size *L_k_* in the function of enrichment score, normalized enrichment score can be calculated, which is the enrichment score divided by the expected value of the corresponding null distribution $F^{G_{k}}=F^{{\bar{G}}_{k}}$.

### 2.2 Multilevel task groups in multitask learning

**Fig. 1.**
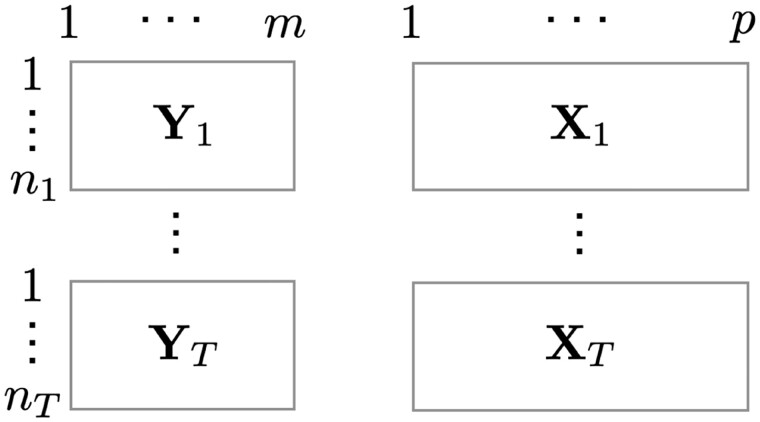
General representation of data for modeling *m* multiple tasks with *T* multiple levels given *p* features

In multitask learning with feature selection, features associated with multiple tasks are often identified. When there are heterogeneous sample groups or levels in each task, we are interested in identifying associated features with each task in each group or level. For example, Han and Zhang (2015) proposed the multilevel task grouping method and Zhao *et al.* (2022) proposed mix-lasso for identifying multitask multilevel features. Let us suppose there are *m* learning tasks, e.g. cancer drugs, *p* features, e.g. genes and each task is measured across *T* heterogeneous sample groups, e.g. drug-treated cell lines of *T* cancer types. In Figure 1, we illustrate the general situation with *T* levels of *m* tasks output data **Y** and *T* levels of *p* features input data **X**. There are a total of $\left(2^{m}-1)\cdot (2^{T}-1\right)$ possible sets of features associated with individual tasks and individual levels.

## 3 Implementation

**Fig. 2.**
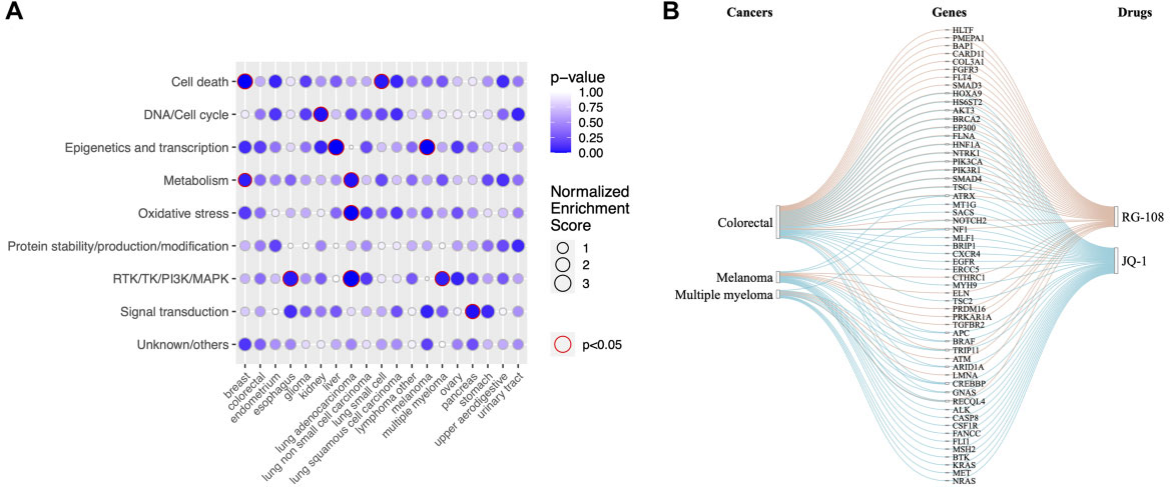
An example of visualizing the results of drug-set enrichment analysis and an Sankey diagram example of visualizing associations of a gene set with two cancer drugs corresponding to three cancer types. (**A**) For each cancer tissue type (*x*-axis), a list of 147 ranked drugs was analyzed for their enrichment among nine drug classes (*y*-axis). (**B**) A Sankey diagram illustrating how a set of 56 genes is associated with two drugs (RG-108 and JQ-1), corresponding to three cancer types (colorectal, melanoma and multiple myeloma). The plot shows the results from a mix-lasso analysis (Zhao *et al.*, 2022) to identify genomic features that are associated with the sensitivity of cell lines from these cancer types to RG-108 or JQ-1 as measured by cell viability assays

*EnrichIntersect* is an R package that generalizes gene set enrichment analysis, so that one can identify over-represented custom sets among any ranked feature list in response to given phenotypes. The enrichment() function requires a scored list of features which is either a vector corresponding to one phenotype or a matrix corresponding to multiple phenotypes, and a data.frame object with user-defined sets for all the features in the scored list. CSEA provides (normalized) enrichment scores for the list of features enriched to multiple custom sets for a single phenotype, for example, multiple drug classes for a given cancer type in one column of Figure 2A. We use red colored circles to visualize statistically significant enriched custom sets at a user-specified significance level with a prespecified multiple-comparison correction method.

To visualize a large number of features associated with multitask multilevel responses, as in Figure 1, *EnrichIntersect* has adapted the *network3D* package for visualizing a large number of intersecting sets at a glance. One intersecting set is a list of features associated with a combination of a subset of tasks and a subset of sample groups or levels. It is not straightforward to visualize all possible $\left(2^{m}-1)\cdot (2^{T}-1\right)$ intersecting sets for *m* tasks and *T* levels. The *network3D* package can visualize only a small or moderate number of features at each stage. Our package *EnrichIntersect* passes suitable parameters to JavaScript in *networkD3* for visualizing a large number of features, small number of tasks and small number of levels, which is useful in many applications. For example, Figure 2B shows a relatively large number of genomic features associated with a few drugs (i.e. tasks) and a few cancer types (i.e. levels). Note that our package requires an array object of the estimated regression coefficients from a multitask multilevel model as an input data for the function intersectSankey().

The R package *EnrichIntersect* has been made freely available and it has passed all the required quality checks and platforms on CRAN (https://CRAN.R-project.org/package=EnrichIntersect). A user-manual description of features, function calls and examples can be found from the vignette of our package on CRAN.

## 4 Conclusion

The *EnrichIntersect* R package provides a flexible tool for enrichment analysis based on user-defined sets. It allows users to perform over-representation analysis of the custom sets among any specified ranked feature list, hence making enrichment analysis applicable to various types of data from different scientific fields. A possible extension of CSEA is to use Fisher’s exact test or hypergeometric test for unranked list of features. *EnrichIntersect* also enables an interactive means to visualize identified associations based on, for example, the multilevel task grouping method (Han and Zhang, 2015), mix-lasso (Zhao *et al.*, 2022) or any other similar method most suitable for particular application scenarios with multitask and multilevel data, as illustrated in Figure 1. It refines the use of the *networkD3* package to allow the visualization of relationships between a large number of features and multiple tasks corresponding to multiple sample groups or levels.

## Data Availability

The data associated with this article are openly available in the R package *EnrichIntersect* at https://CRAN.R-project.org/package=EnrichIntersect.
